# A matched case-control study of risk factors associated with multiple sclerosis in Kuwait

**DOI:** 10.1186/s12883-020-01635-1

**Published:** 2020-02-21

**Authors:** Hadeel El-Muzaini, Saeed Akhtar, Raed Alroughani

**Affiliations:** 1 Department of Community Medicine and Behavioural Sciences, Faculty of Medicine, University of Kuwait, PO Box 24923, 13110 Safat, Kuwait; 2 Division of Neurology, Department of Medicine, Amiri Hospital, Arabian Gulf Street, 13041 Sharq, Kuwait

**Keywords:** Multiple sclerosis, Family history, Matched case-control study, Conditional logistic regression, Middle-east

## Abstract

**Background:**

Genetic and environmental factors seem to have etiologic roles in multiple sclerosis (MS). Kuwait is regarded as medium to high risk country for MS. However, there is a paucity of published data on the risk factors for MS in Kuwait. Therefore, this matched case-control study examined the association between various factors including family history, stressful life events, exposure to tobacco smoke, vaccination history, comorbidities and MS risk in Kuwait.

**Methods:**

Confirmed 110 MS cases and age (± 5 years), gender and nationality matched controls (1:1) were enrolled. A pre-tested structured questionnaire was used to collect the data through face-to-face interviews both from cases and controls. Conditional logistic regression was used to analyze the data.

**Results:**

Among both cases and controls, majority were Kuwaiti (82.7%), and female (76.4%). Multivariable model showed that cases compared to controls were significantly more likely to have had a family history of MS (adjusted matched odds ratio (mOR_adj_) = 5.1; 95% CI: 2.1–12.4; *p* < 0.001) or less likely to have been vaccinated against influenza A and B viruses before MS onset (mOR_adj_ = 0.4; 95% CI: 0.2–0.8; *p* = 0.010). None of the other variables considered were significantly related to MS status in this study.

**Conclusions:**

Family history of MS had significantly direct, whereas, vaccination against influenza A and B viruses had inverse associations with MS status. Future studies may contemplate to verify the observed results.

## Introduction

Multiple sclerosis (MS) is a chronic inflammatory neurological disorder that affects young adults, causing a range of morbidities and disabilities [1–3]. Globally, the estimated number of people with MS has increased from 2.1 million in 2008 to 2.3 million in 2013 [4], with higher prevalence in females [1]. Also, in Kuwait the sex ratio (female: male) of 1.9 among MS cases has been reported [5, 6]. The precise etiology of MS continues to be undefined. However, epidemiological studies have suggested that environmental exposures of genetically susceptible individuals play an important role in the causation of MS. Additionally, family history, ethnicity, viral infections mainly Epstein–Barr virus (EBV), vitamin D deficiency, geographic location have been shown to be the risk factors for MS [1]. Month of birth has been shown to increase MS risk in different countries, which might be associated with varying length of exposure to sunlight and perhaps resulting in vitamin D deficiency [1, 7, 8]. Smoking including its dose and duration have been shown to be associated with increased MS risk [1, 9–12], disease progression [1, 13], and excess mortality among MS patients [14].

Kuwait experienced an increase in MS incidence (per 100,000 population), during 1993 (1.1), 2000 (2.6), 2011 (6.9) and the corresponding MS prevalence (per 100,000 population) of 6.7, 14.8 and 85.1 for these years respectively [15, 16]. Advocated plausible explanations for the increased MS burden in Kuwait include public awareness, socioeconomic status changes, adoption of revised versions of diagnostic criteria, better ascertainment of the cases, increased number of neurologists and availability of magnetic resonance imaging (MRI) and other diagnostic tests, in addition to the introduction of the national MS registry [16]. Moreover, environmental exposures [3, 7], changing lifestyle and dietary patterns of Kuwaiti populations have been implicated [3]. Parenthetically the prevalence of tobacco smoking is very high (20.5%) in Kuwait [17], and practiced both by smoking cigarettes and waterpipe [18]. In Kuwait, a large proportion of adolescents (13–15 years) also get exposed to tobacco smoke through various modes including active cigarette smoking (21.1%), waterpipe use (16.2%) and/ or through exposure to secondhand tobacco smoke (42.9%). However, few studies conducted in Kuwait showed non-significant relationship between tobacco smoking and MS risk [3, 7], which warranted further investigations. Furthermore, concurrent comorbidities and vaccinations against viral infections suspected to contribute to MS risk also need to be explored in the settings of the Middle Eastern countries including Kuwait. Therefore, this matched case-control study was sought to examine the association between various potential risk factors and MS risk in Kuwait.

## Participants and methods

### Study design and setting

A matched case-control design was implemented to address the objective of this study. Cases were enrolled from neurology clinics at Ibn Sina Hospital and MS clinic at Dasman Diabetes Institute. These are the only two neurology clinics which deal with confirmatory diagnosis and treatment of MS cases in Kuwait. Cases suspected for MS seen in any other clinics/ hospitals in Kuwait are referred to one of these two clinics (our study sites) for confirmatory diagnosis and treatment. Subsequently confirmed MS cases are registered in Kuwait National MS Registry. Controls were selected from among the staff and students of the Kuwait University. University staff included academic members, administrative and secretarial staff. This is the only public-sector university in the country, offering free education to all enrolled students. Therefore, it was assumed that faculty, staff and enrolled students represent the typical Kuwaiti population.

### Case definition, inclusion and exclusion criteria

A case was defined as a patient of any age, gender, nationality and resident of Kuwait with a confirmed MS, diagnosed by a neurologist using the revised 2010 McDonald Criteria [19]. Individuals with cognitive impairment or any neurological disease other than MS were excluded. Resident status in Kuwait indicates that the person is not a short-term visitor. Resident status defines that the person is either a Kuwaiti citizen or a migrant worker (with work permit) including dependent(s) of a migrant worker.

### Control definition, inclusion and exclusion criteria

A control was defined as a resident of Kuwait without prior history of confirmed MS diagnosis. Controls were individually matched with each case for age (± 5 years), gender and nationality. Each control was given a date of pseudo-onset of MS which matched with the date of onset of corresponding MS case. Individuals with poor cognitive ability or any past or present neurological disease were excluded.

### Questionnaire development and data collection

A face-to-face interview of each case and his/ her pair matched control was conducted using a structured questionnaire to collect the data on socio-demographic, potential genetic and environmental factors identified through literature review [3, 9]. Enrollment of cases and controls was carried out from July 1, 2016 to September 30, 2016. Date of MS onset was labelled as index year and history of potential exposures were assessed prior to the end of that year. Similarly, in the matched control a date of pseudo-onset of MS was constructed, which is the date of MS onset of the case. Control subjects were asked to report their exposures prior to the end of the index year (i.e. year of their pseudo-onset of MS). Questions on socio-demographic factors included age (completed years), gender, nationality, place of birth, birth order, number of siblings and relationship of parents. Participants were asked about their education level, occupation, family income, marital status and governorate of residence at the time of MS onset, and history of all countries lived in prior to disease. Age (completed years), self-reported height and weight at time of MS onset were recorded. History of vaccination, presence of other disease(s), head trauma, and self-reported vitamin D status was taken. Assessment of genetic and environmental exposures included family history of MS and other diseases including autoimmune diseases (inflammatory bowel disease, systemic lupus erythematosus, rheumatoid arthritis, Graves’ disease), presence in Kuwait during first Gulf war of 1990, history of exposure to solvents and vaccinations’ history. History of exposure to tobacco smoke and secondhand smoking was obtained from cases and controls, by asking about previous smoking status [20, 21]. Exposure to tobacco smoke for the period prior to MS onset/pseudo-onset was assessed both in cases and controls respectively. Data were gathered on average number of cigarettes/waterpipes smoked, frequency and duration of tobacco smoking. Subsequently the amount of tobacco smoked over a period of interest was calculated as pack-years. Data on exposure to secondhand smoking was obtained, by asking participants if they have been exposed to tobacco smoke at home or elsewhere. Approximate duration (years) of secondhand smoking was calculated. The questionnaire was pretested and modified if required before actual use in the study. The final study questionnaire can be found as a Additional files 1 and 2.

### Sample size

By taking into account 10% potential non-response and to achieve a statistical power (1-β) of 80%, this matched case-control study was designed to enroll 100 patients with MS and 100 MS-free matched controls (1:1 ratio) to relate most of the potential exposures (having a prevalence 0.25 or higher in the general population) and MS status with a matched odds ratio (mOR) 2.5 or higher using conditional logistic regression at a 5% significance level (α) [22].

### Ethics

Written consent was obtained from each study participant after explaining the aims and potential benefits of the study, length of questionnaire and time required to answer all the questions. Ethical approval of the study protocol was granted by Health Sciences Center Ethical Committee, Kuwait University and The Standing Committee for Coordination of Health and Medical Research, Ministry of Health, Kuwait.

### Statistical analysis

Descriptive statistics including means and standard deviations of quantitative variables and frequencies (%) of qualitative variables were computed. Variables significantly (*p* ≤ 0.15) related with MS status on univariable conditional logistic regression analysis were considered for their possible inclusion in the final multivariable conditional logistic regression model. The variables significantly (*p* < 0.05) and independently associated with MS status were retained in the final model. Adjusted mOR (mOR_adj_) and their 95% confidence intervals (CI), were calculated from the parameter estimates of the final model. Statistical analyses were performed using Stata 14.2. (College Station, TX: StataCorp LP).

## Results

### Characteristics of case and control samples

**Fig. 1:**
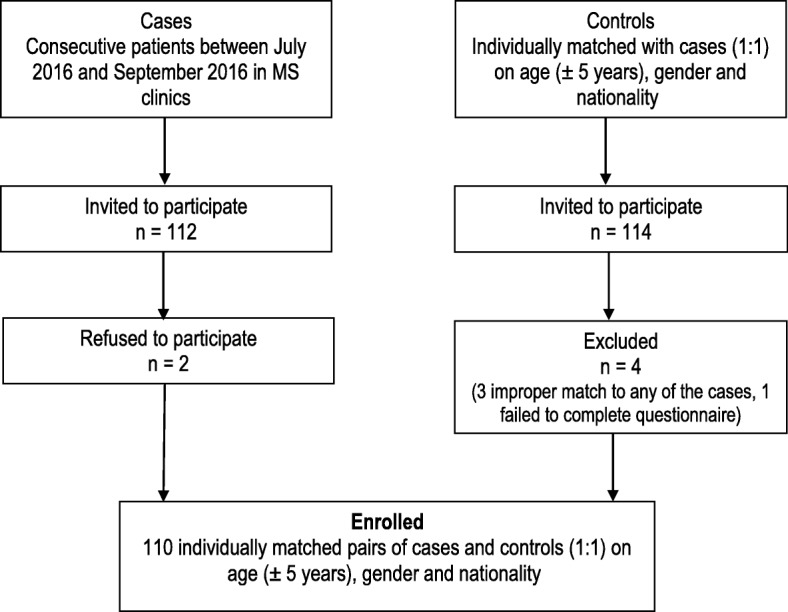
Flow chart describing the enrollment of the MS cases and controls

In this case-control study, 112 MS cases were invited to participate and 110 (98.2%) consented for enrollment. One hundred and ten MS cases and 110 control subjects, individually matched with cases (1:1) on age (± 5 years), gender and nationality were enrolled (Fig. 1). The mean (SD) age (years) was 34.8 (10.3) and 34.9 (10.6) for cases and controls respectively. The mean (SD) age was 27.3 (9.0) years was nearly the same at the time of MS onset in cases or pseudo-onset in controls. Both in cases and controls, 82.7% were Kuwaiti nationals and 76.4% were females. The distributions of demographic characteristics such as marital status, monthly income (KD), governorate of residence and relative distributions of potential risk factors in cases and controls are shown in Tables 1 and 2 respectively.

**Table 1:** Socio-demographic characteristics of cases and controls enrolled in a case-control study of risk factors for multiple sclerosis in Kuwait

Characteristics	MS patients (N_1_ = 110)	Controls (N_2_ = 110)
	n_1_ (%)	n_2_ (%)
Current age (years)		
< 20	8 (7.3)	4 (3.6)
20–29	28 (25.5)	34 (30.9)
30–39	43 (39.1)	40 (36.4)
40–49	19 (17.3)	19 (17.3)
≥ 50	12 (10.9)	13 (11.8)
Mean (SD)	34.8 (10.3)	34.9 (10.6)
Age (years) at MS onset Mean (SD)	27.3 (9.0)	27.3 (9.0)
Gender		
Male	26 (23.6)	26 (23.6)
Female	84 (76.4)	84 (76.4)
Nationality		
Kuwaiti	91 (82.7)	91 (82.7)
Non-Kuwaiti	19 (17.3)	19 (17.3)
Country of birth		
Kuwait	98 (89.1)	103 (93.6)
Abroad	12 (10.9)	7 (6.4)
Marital status at MS onset		
Never married	54 (49.1)	61 (55.5)
Ever married	56 (50.9)	49 (44.5)
Income (KD/month) at MS onset		
≤ 600	13 (12.4)	13 (12.0)
601–1200	25 (23.8)	24 (22.2)
> 1201	67 (63.8)	71 (65.7)
Area of residence		
Capital	30 (27.5)	22 (20.0)
Hawalli	36 (33.0)	53 (48.2)
Farwaniya	13 (11.9)	7 (6.4)
Ahmadi	8 (7.3)	11 (10.0)
Jahra	4 (3.7)	7 (6.4)
Mubarak Al Kabeer	18 (16.5)	10 (9.1)

**Table 2:** Distribution of potential risk factors in cases and controls enrolled in a case-control study of risk factors for multiple sclerosis in Kuwait

Characteristics	MS patients (N_1_ = 110)	Controls (N_2_ = 110)
	n_1_ (%)	n_2_ (%)
BMI		
< 25	50 (50.0)	54 (52.4)
25–29.9	28 (28.0)	28 (27.2)
≥ 30	22 (22.0)	21 (20.4)
Education level at MS diagnosis (≤ high school vs. > high school level)	29 (26.4)	19 (17.3)
Birth order (1st/2nd or more)	24 (21.8)	26 (23.6)
Parents’ relationship		
Unrelated	80 (72.7)	73 (66.4)
First degree cousins	22 (20.0)	21 (19.1)
Second degree cousins	8 (7.3)	16 (14.5)
Presence during Gulf war (yes/no)	59 (53.6)	71 (64.5)
Family history of MS (yes/no)	33 (30.0)	13 (11.8)
Tobacco smoking (yes/no)	18 (16.4)	12 (10.9)
Childhood exposure to environmental tobacco smoke (yes/no)	47 (42.7)	56 (50.9)
Regular passive smoking (yes/no)	67 (62.0)	69 (62.7)
Tonsillectomy before MS onset (yes/no)	10 (9.1)	9 (8.2)
Appendectomy before MS onset (yes/no)	9 (8.2)	3 (2.7)
Receiving Influenza vaccine before MS onset (yes/no)	18 (16.4)	32 (29.1)
Self-reported vitamin D deficiency (yes/no)	83 (75.5)	76 (69.1)

### Univariable conditional logistic regression analyses

Univariable conditional logistic analyses showed that cases were significantly more likely to have positive family history of MS (*p* = 0.002) or less likely to receive influenza virus A and B vaccine (*p* = 0.030). However, cases compared to their matched controls were not statistically significantly different in terms of active or passive tobacco smoking, number of siblings, parent’s relationship, presence in Kuwait during first Gulf war of 1990, history of tonsillectomy and appendectomy. Also, distributions of self-reported vitamin D deficiency, area of residency, history of chickenpox, mumps and measles infections, regular exposure to solvents, vaccination status against hepatitis B virus and measles-mumps-rubella viruses were not statistically significantly different between MS cases and their matched controls (Table 3).

**Table 3:** Univariable conditional logistic regression analyses of risk factors associated with multiple sclerosis in a case-control study in Kuwait

Variables^a^	Matched OR	95% CI	*p*-value
Country of birth (Kuwait vs. abroad)	3.5	0.7–16.8	0.118
Education level at MS onset (≤ high school vs. > high school)	2.7	1.0–6.8	**0.040**
Marital status at MS onset (ever vs. never)	1.5	0.8–2.9	0.240
Household monthly income (KD)			
≤ 600	1.0	–	
601–1200	1.0	0.4–2.8	0.959
> 1200	0.8	0.3–2.1	0.703
BMI			
< 25	1.0	–	
25–29.9	1.0	0.5–2.1	0.964
≥ 30	1.2	0.6–2.7	0.596
Birth order (2nd or more vs. 1st)	1.1	0.6–2.2	0.732
Parents relationship			
Un-related	1.0	–	
First degree cousins	0.9	0.5–1.9	0.862
Second degree cousins	0.5	0.2–1.2	0.109
Presence during Gulf war (yes vs. no)	0.5	0.3–1.0	0.062
Family history of MS (yes vs. no)	3.5	1.6–7.7	**0.002**
Tobacco smoking status (yes vs. no)	2.2	0.8–6.3	0.144
Childhood exposure to environmental tobacco smoke (yes vs. no)	0.7	0.4–1.2	0.210
Regular passive smoking (yes vs. no)	1.0	0.5–1.8	0.876
Tonsillectomy before MS onset (yes vs. no)	1.1	0.4–2.9	0.808
Appendectomy before MS onset (yes vs. no)	3.0	0.8–11.1	0.099
Receiving Influenza vaccine before MS onset (yes vs. no)	0.5	0.2–0.9	**0.030**
Self-reported vitamin D deficiency (yes vs. no)	1.4	0.8–2.6	0.277

^a^Distribution of area of residence, number of siblings, chicken pox, mumps and measles infections, receiving Hepatitis B virus vaccine and measles-mumps-rubella vaccine, regular exposure to solvents and history of autoimmune diseases (i.e. inflammatory bowel disease, systemic lupus erythematosus, rheumatoid arthritis, Graves’ disease) were not significantly different between MS cases and their matched controls

### Multivariable conditional logistic regression model

Final multivariable conditional logistic regression model showed that after adjusting for the effect of educational level, the cases compared to controls were more likely to have had a positive family history of MS (mOR_adj_ = 5.1; 95% CI: 2.1–12.4; *p* < 0.001). Additionally, after taking into account the effect of educational level, cases compared to control were significantly less likely to have had received influenza virus A and B vaccine before MS onset (mOR_adj_ = 0.4; 95% CI: 0.2–0.8; *p* = 0.010) (Table 4).

**Table 4:** Multivariable conditional logistic regression model of risk factors associated with multiple sclerosis in a case-control study in Kuwait

Risk factor	Adjusted matched OR^a^	95% CI	*p*- value
Family history of MS (yes vs. no)	5.1	2.1–12.4	< 0.001
Influenza vaccination (yes vs. no)	0.4	0.2–0.8	0.010

^a^Model is adjusted for ‘education level” i.e. ≤ high school vs. > high school

## Discussion

This matched case-control study examined the association of various risk factors and MS risk in Kuwait. The multivariable conditional logistic regression model showed that family history of MS was significantly and independently associated with MS in this study. Family history is an established risk factor for MS [1, 3, 7, 11], seemingly through enhanced susceptibility resulting from gene-gene interactions in human leukocyte antigen (HLA) system [11, 23]. While a clear association with variation in HLA has been established in MS, which explains up to 10.5% of the genetic variance of underlying risk, other genetic regions beyond HLA also have been shown to enhance the susceptibility to MS [24]. In multivariable analysis, vaccination against influenza A and B viruses showed a protective effect against MS risk. To the best of our knowledge, a non-specific protective effect of influenza A and B viruses’ vaccine against MS development has not been shown previously. This association possibly could be coincidental with no biological causal link [25]. Nonetheless, observations of autoimmune diseases in animal models suggest that some infections may enhance the risk while others may contribute to prevent autoimmune diseases, showing diversity in the host immune response [26]. Furthermore, it is difficult to assess the effect of influenza vaccine on MS risk since the antigenic composition of seasonal influenza vaccines changes annually depending upon the prevalent influenza virus serotypes [27]. Further studies are needed to better understand the role of influenza vaccine in MS risk observed in this study.

The findings of this study revealed that tobacco smoking did not show a statistically significant association (active, and/or environmental exposure to tobacco smoke) with MS status. The results of this study are consistent with those of few earlier studies conducted in Kuwait, which did not reveal significant relationship between tobacco smoking and MS risk [3, 7, 28]. However, elsewhere, tobacco smoking has been shown repeatedly as a significant risk factor for increased MS risk [1, 9–12]. Furthermore, it has been previously shown that the effect of tobacco smoke on MS risk is genetically-dependent, and genetic compositions vary across the populations [9, 29, 30]. A concurrent and/ or alternate explanation may be that tobacco smoking both active and passive is highly prevalent in general population in Kuwait that posed us a difficulty in segregation and measuring the exposure to active tobacco smoking and to environmental tobacco smoke. Future studies should consider assessing the exposure to tobacco smoke by cotinine testing (saliva or urine samples) of both cases and controls in Kuwait.

Several variables were not statistically significantly associated with MS status such as country of birth, area of residence, level of education, marital status, socioeconomic status and BMI at time of disease onset, number of siblings, birth order and degree of relationship of parents. Some other previously reported important risk factors for MS including regular exposure to solvents, history of tonsillectomy, appendectomy and self-reported vitamin D deficiency [1, 10], did not attain statistical significance either because these factors indeed did not pose a substantial MS risk in our setting or probably due to low study power regarding these exposures, thus need further evaluation.

We also did not find a significant association between hepatitis B virus and measles-mumps-rubella vaccination status. These findings are consistent with those reported in systematic reviews of relationship between MS onset and vaccination against several infections such as measles-mumps-rubella, hepatitis B virus, influenza virus, human papilloma virus, diphtheria, tetanus, pertussis, or meningococcal disease [25, 27].

Few limitations of this study should be considered. First, recall bias is an inherent limitation of case-control study design, cases might have remembered exposure to various risk factors better than the controls. However, the time window for ascertaining exposures was the same for matched controls and cases. Secondly, the possibility of under-reporting of smoking among MS patients especially females, which could be driven by feeling of guilt or social implications of admitting smoking in the presence of a relative or family member. Thirdly, smoking is less common among females, whom composed most cases, therefore a larger sample size might be needed to assess the association between smoking and MS risk.

## Conclusions

This study showed that family history of MS was significantly associated with an increased MS risk, whereas, vaccination against influenza A and B viruses offered significant protection against MS. Further studies are indicated to verify this observed and previously un-reported association. Furthermore, question remained unanswered is why tobacco smoking continues to have a non-significant relationship with MS in this and previous studies reported from Kuwait as opposed to what has been consistently reported elsewhere. Future studies need to consider this aspect perhaps through objective measurement of exposure to tobacco smoke in the Middle Eastern countries including Kuwait.

## Supplementary information

**Additional file 1.** Questionnaire for Case

**Additional file 2.** Questionnaire for Control

## Data Availability

The datasets used and analysed during the current study are available from the corresponding author on reasonable request.

## Abbreviations

CI: Confidence intervals
EBV: Epstein–Barr virus
mOR: Matched odds ratio
mOR_adj_: Adjusted mOR
MRI: Magnetic resonance imaging
MS: Multiple sclerosis
