# Correction to: A matched case-control study of risk factors associated with multiple sclerosis in Kuwait

**DOI:** 10.1186/s12883-020-01886-y

**Published:** 2020-08-19

**Authors:** Hadeel El-Muzaini, Saeed Akhtar, Raed Alroughani

**Affiliations:** 1 Department of Community Medicine and Behavioural Sciences, Faculty of Medicine, University of Kuwait, PO Box 24923, 13110 Safat, Kuwait; 2 Division of Neurology, Department of Medicine, Amiri Hospital, Arabian Gulf Street, 13041 Sharq, Kuwait

**Correction to: BMC Neurol 20, 64 (2020)**

**https://doi.org/10.1186/s12883-020-01635-1**

Following publication of the original article [1], the authors reported an error in Table 3. The correct version of the table can be seen below, wherein the added data are set in italic and that table footnote ‘Hepatitis B virus vaccine’ was removed.

**Table 3:** Univariable conditional logistic regression analyses of risk factors associated with multiple sclerosis in a case-control study in Kuwait

Variables^a^	Matched OR	95% CI	*p*-value
Country of birth (Kuwait vs. abroad)	3.5	0.7–16.8	0.118
Education level at MS onset (< high school vs. high school or higher)	2.7	1.0–6.8	**0.040**
Marital status at MS onset (ever vs. never)	1.5	0.8–2.9	0.240
Household monthly income (KD)			
≤ 600	1.0	–	
601–1200	1.0	0.4–2.8	0.959
> 1200	0.8	0.3–2.1	0.703
BMI			
< 25	1.0	–	
25–29.9	1.0	0.5–2.1	0.964
≥ 30	1.2	0.6–2.7	0.596
Birth order (2nd or more vs. 1st)	1.1	0.6–2.2	0.732
Parents relationship			
Un-related	1.0	–	
First degree cousins	0.9	0.5–1.9	0.862
Second degree cousins	0.5	0.2–1.2	0.109
Presence during Gulf war (yes vs. no)	0.5	0.3–1.0	0.062
Family history of MS (yes vs. no)	3.5	1.6–7.7	**0.002**
Tobacco smoking status (yes vs. no)	2.2	0.8–6.3	0.144
Childhood exposure to environmental tobacco smoke (yes vs. no)	0.7	0.4–1.2	0.210
Regular passive smoking (yes vs. no)	1.0	0.5–1.8	0.876
Tonsillectomy before MS onset (yes vs. no)	1.1	0.4–2.9	0.808
Appendectomy before MS onset (yes vs. no)	3.0	0.8–11.1	0.099
Receiving Influenza vaccine before MS onset (yes vs. no)	0.5	0.2–0.9	**0.030**
*Receiving Hepatitis B virus vaccine before MS onset (yes* vs. *no)*	*0.3*	*0.1–0.6*	***0.001***
Self-reported vitamin D deficiency (yes vs. no)	1.4	0.8–2.6	0.277

^a^Distribution of area of residence, number of siblings, chicken pox, mumps and measles infections, receiving measles-mumps-rubella vaccine, regular exposure to solvents, and history of autoimmune diseases (i.e. *inflammatory bowel disease*, *systemic lupus erythematosus*, *rheumatoid arthritis*, *Graves’ disease*) were not significantly different between MS cases and their matched controls
