# Supplementary material for: A matched case-control study of risk factors associated with multiple sclerosis in Kuwait
Source: BMC Neurol. 2020 Feb 21;20:64. doi: 10.1186/s12883-020-01635-1 (PMC7033919; doi:10.1186/s12883-020-01635-1)
Supplement: Supplementary file 1 — Additional file 1. Questionnaire for Case [file 12883_2020_1635_MOESM1_ESM.docx]

| [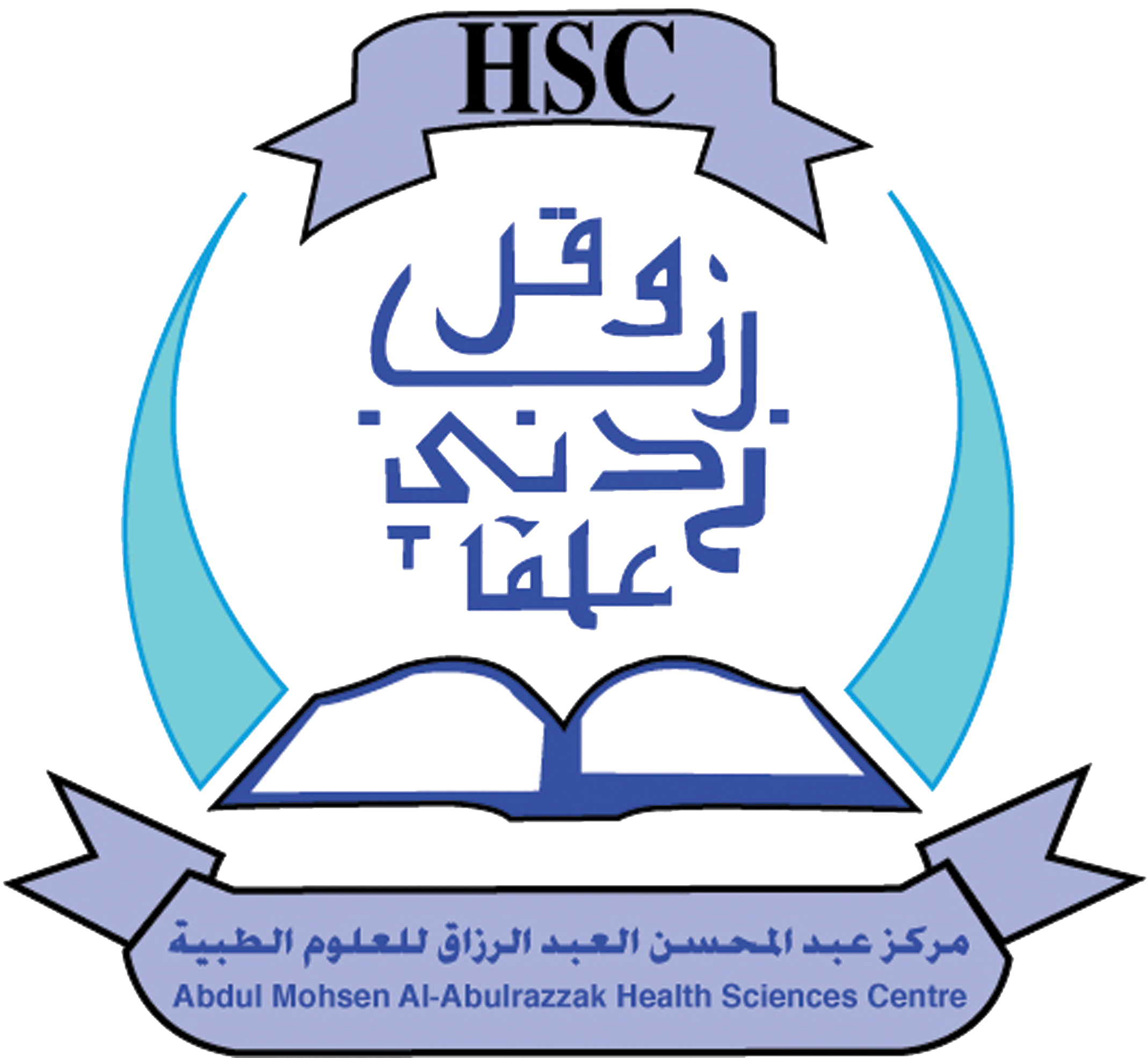](https://www.google.com.kw/url?sa=i&rct=j&q=&esrc=s&source=images&cd=&cad=rja&uact=8&ved=0ahUKEwjola_tq9fLAhUDcRQKHZwDDNMQjRwIBw&url=http://www.hsc.edu.kw/ICH/Contact.aspx&psig=AFQjCNHxSjvIcxPUqc_WVFmhZBdLoz452g&ust=1458840855358874) | Kuwait UniversityFaculty of MedicineMPH Program |
| --- | --- |

# Risk factors associated with multiple sclerosis in Kuwait- Case

Please take a few minutes to fill out this questionnaire.

I welcome your feedback and your answers will be kept confidential. Thank you for your participation.

## Questions

|  |  |  |  |  |  |  |  |
| --- | --- | --- | --- | --- | --- | --- | --- |

### What is your date of birth? dd|mm|yyyy

### What is your gender? _0_ Male _1_ Female

### What is your place of birth (city-country)? ….…………….………… - ……………………………..

### What is your nationality? _1_ Kuwaiti _0_ Non-Kuwaiti Specify……………………………

### What is your blood group?

### _1_ O _2_ A _3_ B _4_ AB _5_ Don’t know

1. What is your rhesus (Rh) factor? _1_ Positive _2_ Negative _3_ Don’t know

### Are your parents related?

### _0_ No

_1_ First degree (cousins)

_2_ Second degree (grandparents are cousins)

_3_ Third degree (parents of grandparents are cousins)

1. How many members were in your family (including yourself)?

…………. Males ………….. Female

1. What is your birth order among siblings? …………………………………

### Were you in Kuwait during the Iraqi invasion 1990-1991? _1_ Yes _0_ No

### If yes, were you exposed to any of the following?

### Imprisonment _1_ Yes _0_ No

### Beating/torture _1_ Yes _0_ No

### Hiding _1_ Yes _0_ No

### Toxic fumes from burning oil wells _1_ Yes _0_ No

1. Were any of your family members ever diagnosed with MS? _1_ Yes _0_ No
2. If yes, how are they related to you? …………………………………………
3. Do you have any neurological disease other than MS? _1_ Yes _0_ No

### At diagnosis, what was your:

### Age…………..completed years Weight ……..…Kg Height ….……cm

1. At diagnosis, what was your marital status?

_1_ Single  _2_ Married  _3_ Divorced

_4_ Widowed

1. At diagnosis, what was your level of education?

_1_ Illiterate  _2_ Read and write  _3_ Primary school

_4_ Secondary school  _5_ High school  _6_ University and above

1. At diagnosis, what was your occupation?

_1_ Student  _2_ Employed  _3_ Retired

_4_ Housewife  _5_ Other ……………………………………………….

1. At diagnosis, what was your family monthly income (KD)?

_1_ Up to 350  _2_ 351 - 600  _3_ 601 - 1200

_4_ 1201 - 2000  _5_ >2000

1. At diagnosis, in which governorate did you live?

_1_ Capital  _2_ Hawalli  _3_ Farwaniya

_4_ Ahmadi  _5_ Jahra  _6_ Mubarak Al-Kabeer

1. List all the geographic locations where you spent more than six months (starting from birth till time of diagnosis).

| **Town** | **Country** | **From year** | **To year** |
| --- | --- | --- | --- |
|  |  |  |  |
|  |  |  |  |
|  |  |  |  |
|  |  |  |  |
|  |  |  |  |
|  |  |  |  |
|  |  |  |  |

### Before diagnosis, did you regularly smoke cigarettes/ waterpipe or any other tobacco product?

### _1_ Yes _0_ No (if no proceed to question 26)

### Before diagnosis, on average, how many of the following products did you smoke each (day/week)?

_1_ Number of manufactured cigarettes |__|__|__| per day / week duration……………….years

_2_ Number of waterpipe sessions |__|__|__| per day / week duration………..……. years

_3_ Pipes of tobacco |__|__|__| per day / week duration………..……. years

_4_ Cigar, cheroots, or cigarillos |__|__|__| per day / week duration………..……. years

_5_ No of hand rolled cigarettes |__|__|__| per day / week duration………..……. years

_6_ Any others, specify………………………………. |__|__|__| per day / week duration…………….years

### At which age did you start smoking? ……………………….years

### If you quit smoking, at which age was that? ……………………….years

### When your mother was pregnant with you, did either of your parents smoke?

_0_ No  _1_ Father  _2_ Mother  _3_ Don’t know

### When you were a child, did anyone regularly smoke inside your home? _1_ Yes _0_ No

### Before the age of……years old, as an adult, have you ever lived with anybody who smoked in your home or worked in places where people smoked around you?

_0_ No  _1_ 1-5 years  _2_ 5-10 years

_3_ 11-20 years  _4_ More than 20 years

### Before diagnosis, how much time did you spend in public areas where people smoked around you (e.g. café, diwaniya, family gatherings)?

_0_ Less than 3 hours/week  _1_ 3-6 hours /week  _2_ 7-9 hours/week

_3_ More than 10 hours/week

### Before diagnosis, were you exposed to incense at any of the following places: home, work or social gathering?

_0_ Rarely  _1_ Less than once/week  _2_ Once/week

_3_ 3-5 times/week  _4_ Daily

1. Before diagnosis, were you frequently exposed to solvents (paints, pesticides, herbicides)?

_1_Yes  _0_No

### Before diagnosis, did you have any of the following infections/diseases?

### Measles _1_Yes _0_ No _2_ Don’t know

### Mumps _1_Yes _0_ No _2_ Don’t know

### Chicken pox _1_Yes _0_ No _2_ Don’t know

### Infectious mononucleosis _1_Yes _0_ No _2_ Don’t know

### Tuberculosis _1_Yes _0_ No _2_ Don’t know

### Head trauma _1_Yes _0_No

### Rheumatoid arthritis _1_Yes _0_ No

### Inflammatory Bowel Disease _1_Yes _0_ No

### Systemic Lupus Erythematosus _1_Yes _0_ No

### Grave's disease (Thyroid) _1_Yes _0_ No

### Early onset diabetes (Type 1) _1_Yes _0_ No

### Migraine _1_Yes _0_ No

### Anesthesia _1_Yes _0_No

### Tonsillectomy _1_Yes _0_No Age……….years

### Appendectomy _1_Yes _0_No Age……….years

### Before diagnosis, did you receive any of the following vaccines?

### Hepatitis B _1_Yes _0_No _2_ Don’t know

### MMR (mumps, measles, rubella) _1_Yes _0_No _2_ Don’t know

### Influenza _1_Yes _0_No _2_ Don’t know

### Did any of your family members have any of the following diseases?

### Rheumatoid arthritis _1_Yes _0_ No Relation to you…………….

### Inflammatory Bowel Disease _1_Yes _0_ No Relation to you…………….

### Systemic Lupus Erythematosus _1_Yes _0_ No Relation to you…………….

### Grave's disease (Thyroid) _1_Yes _0_ No Relation to you…………….

### Early onset Diabetes (Type 1) _1_Yes _0_ No Relation to you…………….

### Migraine _1_Yes _0_ No Relation to you…………….

### Before diagnosis, how frequently did you consume dairy products (milk, cheese, yogurt, laban, labnah, ghee, and butter)?

_0_ Less than once/week  _1_ Once/week  _2_ 2-5 times/week

_3_ Once/day  _4_ More than once/day

### Before diagnosis, how frequently did you consume red meat and red meat products?

_0_ Less than once/week  _1_ Once/week  _2_ 2-5 times/week

_3_ Once/day  _4_ More than once/day

### Before diagnosis, how frequently did you consume fish and fish products?

_0_ Less than once/week  _1_ Once/week  _2_ 2-5 times/week

_3_ Once/day  _4_ More than once/day

### Before diagnosis, on average, how long were you exposed to sunshine every day during *summer*?

_0_ Less than 1 hour  _1_ 1-2 hours  _2_ 2-3 hours

_3_ More than 3 hours

### Before diagnosis, on average, how long were you exposed to sunshine every day during *winter*?

_0_ Less than 1 hour  _1_1-2 hours  _2_ 2-3 hours

_3_ More than 3 hours

### Where you ever told by a doctor that you are vitamin D deficient? _1_ Yes _0_ No

**Thank you for taking the time to fill out our study questionnaire.**
